# Familiarity with children improves the ability to recognize children’s mental states: an fMRI study using the Reading the Mind in the Eyes Task and the Nencki Children Eyes Test

**DOI:** 10.1038/s41598-020-69938-4

**Published:** 2020-07-31

**Authors:** Jan Szczypiński, Anna Alińska, Marek Waligóra, Maciej Kopera, Aleksandra Krasowska, Aneta Michalska, Hubert Suszek, Andrzej Jakubczyk, Marek Wypych, Marcin Wojnar, Artur Marchewka

**Affiliations:** 1 Laboratory of Brain Imaging (LOBI), Nencki Institute of Experimental Biology, Polish Academy of Sciences, Pasteur 3, 02-093 Warsaw, Poland; 2 Department of Psychiatry, Medical University of Warsaw, Warsaw, Poland; 3 Laboratory of Neuroinformatics, Nencki Institute of Experimental Biology, Polish Academy of Sciences, Warsaw, Poland; 4 Faculty of Psychology, University of Warsaw, Warsaw, Poland; 5 Department of Psychiatry, University of Michigan, Ann Arbor, MI USA

**Keywords:** Cognitive neuroscience, Emotion, Social neuroscience, Neuroscience

## Abstract

Theory of mind plays a fundamental role in human social interactions. People generally better understand the mental states of members of their own race, a predisposition called the own-race bias, which can be significantly reduced by experience. It is unknown whether the ability to understand mental states can be similarly influenced by own-age bias, whether this bias can be reduced by experience and, finally, what the neuronal correlates of this processes are. We evaluate whether adults working with children (WC) have an advantage over adults not working with children (NWC) in understanding the mental states of youngsters. Participants performed fMRI tasks with Adult Mind (AM) and Child Mind (CM) conditions based on the Reading the Mind in the Eyes test and a newly developed Nencki Children Eyes test. WC had better accuracy in the CM condition than NWC. In NWC, own-age bias was associated with higher activation in the posterior superior temporal sulcus (pSTS) in AM than in CM. This effect was not observed in the WC group, which showed higher activation in the pSTS and inferior frontal gyri in CM than in AM. Therefore, activation in these regions is required for the improvement in recognition of children’s mental states caused by experience.

## Introduction

Humans are social beings, and therefore, the ability to understand the mental states of others is crucial to everyday life and to adequately function in modern society, especially regarding social interactions. This ability is called theory of mind (TOM) or mentalizing. TOM allows us to accurately understand and predict the goals, beliefs, desires and emotions (together described as mental states) of others^1–3^. TOM can be divided into the socio-perceptual component, referring to decoding or detecting other’s mental states based on perceptual information (e.g. a photograph of the eye region) and the socio-cognitive component allowing to infer about others’ intentions or beliefs based on their behaviour and one’s knowledge about the world^4,5^. Another distinction described in the literature divides TOM into affective and cognitive TOM^6^. Affective TOM refers to an ability to infer about feelings of others, while cognitive TOM allows inferring about beliefs.

Several studies showed the own-race bias in socio-perceptual component of TOM^7–9^—a tendency to better recognize mental states of members of the same race. At the same time, the own-race bias could also be observed in less complex processes, such as face recognition (correctly recalling and matching known vs unknown faces)^10^. A similar phenomenon, called the own-age bias, causes the attention to focus on faces of people the same age^11–13^ and better remember those^14,15^. Interestingly, due to experience, ward nurses^16^ and teachers^17^ can become better at remembering the faces of children than control groups can. However, it is currently unknown whether own-age bias affects the ability to understand mental states and, if so, whether this bias can be reduced by experience with other age groups.

An experimental task widely used to measure socio-perceptual component of TOM is the Reading the Mind in the Eyes Task (RMET)^18,19^. There is also a debate on whether RMET engages affective, cognitive or both of these TOM components^18–21^. RMET consists of pictures of the eye region of Caucasian adults paired with adjectives describing mental states (such as playful, amused, and interested). The task requires the processing of the part of the face and gaze direction to choose the correct adjective. RMET was designed specifically to study autism spectrum disorder, however, it was also successfully used in studies of the general population and was later adapted to use in functional magnetic resonance imaging (fMRI). In fMRI studies, the RMET task (Adult Mind condition, AM) is contrasted with a sex recognition task (Adult Sex condition, AS) to obtain brain activations related solely to mindreading. This comparison evokes strong activation in the inferior frontal gyrus (IFG) and posterior superior temporal sulcus (pSTS)^7,22,23^. The IFG has been linked to the human mirror neuron system and observing the actions of others^24^. Facial expressions are also an action type^25^, and the activity of the IFG in the AM condition may reflect facial mimicry and the simulation of others’ facial expressions and mental states^26–28^. Among other functions, the pSTS is involved in decoding social stimuli such as faces and eye gaze^29,30^. Specifically, this region shows higher activation to socially relevant stimuli than socially irrelevant stimuli^29^. It was also proposed that it serves a more general function of the temporal integration of information flow during TOM processing^24^.

Using the original and the East Asian RMET, Adams et al.^7^ showed that white American and Japanese individuals were better at understanding mental states of people of their own race group. When directly comparing AM to AS within a race to the same contrasts in other races, in the case of both groups, increased activation in the pSTS was observed. Interestingly, lower activation in the pSTS in the AM condition for other races was related to the behavioural effect of own-race bias (better accuracy in the own-race AM condition than in the other-race AM condition). Thus, the activity of the pSTS is sensitive to ethnic group membership and corresponds to the behavioural effect of own-race bias. Nevertheless, the own-group advantage can be reduced by experiencing other cultures. For example, in a group of East Asian residents of Canada, performance in the Caucasian RMET was positively associated with the amount of time the participants had lived in Canada, their experience interacting with Caucasians and a shift from their own cultural values towards Canadian values^8^. Similarly, Anatolian Dutch and Moroccan Dutch people were equally good at understanding the mental states of their own ethnic group as they were those of Caucasians^9^. These results indicate the occurrence of the own-race bias in TOM processes and that experience with other cultures can reduce this bias and improve the understanding of mental states of people from other cultural groups.

## Current study

Current evidence shows that people who work with children are better at recognizing children’s faces than control groups^16,17^. This effect can be explained as perceptual expertise acquired by daily contact with children or increased motivation to attend to children faces^24^. Remembering faces was shown to be a predictor of TOM abilities, measured by understanding mental states from the eye region, voice and videos^21^. Therefore, adults who work with children could potentially become experts in understanding the mental states of children. This idea is supported by the fact that people who live in multi-ethnic societies can improve their ability to understand the mental states of people of other ethnic groups^8,9^.

This study aimed to investigate whether a similar effect of experience occurs in adults who work with children. To address this issue, we recruited young childless adults who had a history of working with children or who were working with children at the time of the study (WC) and a second group of childless adults who had no history of working with children (NWC). To measure participants’ ability to understand the mental states of children, we designed the Nencki Children Eyes Test (NCET), which is a test analogous to the RMET but comprises photos of children. We hypothesized that the RMET and NCET would evoke activation in brain areas engaged in mental state processing, specifically the pSTS and IFG, in all participants. We hypothesized that WC would perform better than NWC in the NCET due to their experience working with children. Because decreased pSTS activity seen when trying to understand other-race mental states was related to own-race bias in previous work^7^, we hypothesized that NWC would show a similar decrease in pSTS activity when performing the NCET, which would represent own-age bias in TOM processing, in contrast, we hypothesized that WC would be characterized by increased activation in the pSTS during the NCET, representing a reduction in the own-age bias.

## Methods

### Participants

Thirty-eight healthy, childless adults (age M = 24.08; SD = 3.33) took part in the study: 19 (10 females) who were working with children at the time of the study or who had worked with them in the past for more than half a year and 19 (10 females) who had never worked with children or who had worked with them for less than half a year. The weekly number of hours the WC group spent working with children ranged from 3.5 to 37.5 (M = 14.31; SD = 11.53). The professions of the participants were varied and included school and preschool teachers, sports instructors, babysitters, children’s physiotherapists, and camp counsellors. All participants were of Caucasian ethnicity. Participants were recruited via advertisements in various groups on Facebook. They were mostly students of Warsaw Universities, and all were native Polish speakers.

Subjects signed an informed consent form and were told about the possibility of resigning from further participation at any point of the study. Financial gratification in the amount of 100 PLN (approximately 20–25 EUR) was provided to each subject. The Committee for Research Ethics of the University of Warsaw approved the experimental protocol of the study. The experiment was conducted in compliance with the American Psychological Association’s (APA) Ethical Principles of Psychologists and Code of Conduct (https://www.apa.org/ethics/code/).

### Experimental tasks

#### Reading the Mind in the Eyes Task (RMET)

The revised version of the *RMET*^18^ was designed to measure the ability of the participant to attribute complex mental states (feelings/thoughts/intentions) to others. It consists of 36 photos of the eye region of adults, where each photo is paired with 4 adjectives describing mental states. A participant is asked to choose one term that matches the internal state of the person in the photo. The original task has been previously adapted to the requirements of fMRI studies by limiting the number of displayed adjectives to two instead of four^7^. The sex recognition task was used as a control condition, in which a participant was asked to specify the sex of the person in the photo. For the Polish version of the *RMET,* adjectives were translated from English to Polish by one translator and then back-translated by another translator.

#### Nencki Children Eyes Test (NCET)

Due to the lack of a tool analogous to the *RMET* comprising photos of the eye region of children, we created the *NCET* with 36 photos of the eye region of boys (18) and girls (18) of Caucasian ethnicity. Photos were taken from www.flickr.com, all under the license of noncommercial use with modifications allowed (CC BY-NC 2.0). They were cropped to the eye region, and the colour was changed to black and white. Then, the luminosity and contrast were adjusted using Photo Pos Pro software (https://www.photopos.com/PPP3_BS/Default.aspx) for each image to improve the consistency between the photos^31^.

First, 20 participants (10 females) assessed the sex of the child in the photo, and only photos with 70% accuracy or higher were used in the next step. This step was performed to create a control condition for the fMRI procedure, similar to the control condition for the *RMET* fMRI adaptation^7^. Next, two independent judges ascribed four terms (one correct, three false) describing the mental state expressed by the child in the photo. Afterwards, a new cohort of subjects (n = 20, 10 females) had to choose the most accurate term describing every image. The photo with the adjectives was included only when one term was chosen by at least 50% of the participants (further treated as the correct one), and each of the three others had no more than 25% answers. The procedure was similar to that described by Baron-Cohen et al.^18^ and was repeated until the number of photos in the test did not reach 36. For the purpose of *NCET* fMRI adaptation, we used two adjectives—the one that was the most frequently selected (treated as the correct answer) and the one that was the least frequently chosen in the validation process. Detailed information about the luminance, contrast, and entropy of the stimuli used in *NCET* are presented in Supplementary Table S1. All images used in the NCET are freely available to the scientific community for non-commercial use (https://osf.io/4mxa6/).

### Control tests

Here, we employed various tests to control for possible differences in the studied groups^18,32,33^. We gathered measures of the ability to reason about beliefs, basic emotion recognition, vocabulary knowledge and empathy. Additional information regarding the correlations between the control tests and the NCET and the RMET is provided in Supplementary Table S2.

#### PENN ER-40

The *PENN ER-40* task^34^ was used to assess the ability to recognize basic emotions. This task is performed on a computer and comprises 40 photos of faces, each of which is displayed with five terms, four describing basic emotions—“happy”, “sad”, “angry”, “fear”—and the fifth describing a neutral expression—“neutral”. The participant has to choose which emotion is expressed by the person in the image and then assess how certain they are of their answer on a scale between 0 and 100. The overall score is the sum of the right answers (maximum 40).

#### Hinting task

The *Hinting Task* is a false-belief type of test that was used to measure *TOM*. During this task^35,36^, a participant is presented with ten stories picturing interactions between two persons. Each story ends with a statement stated by one (X) of the two characters, and a question is posed: “What does X truly want to say?”. The participant provides an answer for which 0, 1 or 2 points can be given, according to the solution key. If the answer is rated with 0 points, a hint is presented to the participant, who can provide an additional answer. After being provided with a hint, a participant can obtain only 0 points or 1 point. Participants’ answers are written down, and the points earned and the number of hints given are summed.

#### Comprehension of words test standard version (TRS-S)

*TRS-S* consists of 32 items and is a test of synonyms, i.e., a person has to choose a synonym of a given word from five possible answers, and the maximum score that can be obtained in the test is 32. This test measures vocabulary knowledge and is highly correlated with fluid intelligence and other tests of vocabulary knowledge^37^.

#### Interpersonal reactivity index (IRI)

*IRI* is a multidimensional questionnaire designed by Davis^38^ for measuring four different aspects of empathy: Empathic Concern, Personal Distress, Perspective Taking and Fantasy. However, in the Polish adaptation, the Fantasy subscale was excluded due to a weak theoretical background^39^. The Empathic Concern subscale measures feelings of concern and sympathy for others, the Personal Distress subscale measures negative emotions experienced in tense social settings, and the Perspective Taking subscale measures the ability to take another’s perspective (*put oneself in another’s shoes*). Empathic Concern and Personal Distress are correlated with tools used to measure emotional empathy^38^, while Perspective Taking is correlated with tests used to measure cognitive empathy^38^. The Polish version of the IRI consists of 28 statements (11 for Empathic Concern, 8 for Personal Distress and 9 for Perspective Taking). A participant is asked to mark how he/she agrees with each statement on a 1–5 Likert-type scale ranging from “Completely disagree” to “Absolutely agree”.

### Procedure

#### Behavioural measures

Prior to the fMRI procedure, demographic data were gathered, and behavioural tests described above were administered to subjects in a paper form. The Hinting Task was read aloud by the investigator, and the participant’s answers were written down. Subsequently, the PENN task was completed on a computer. This part of the procedure took approximately 45 min.

#### fMRI procedure

**Figure 1:**
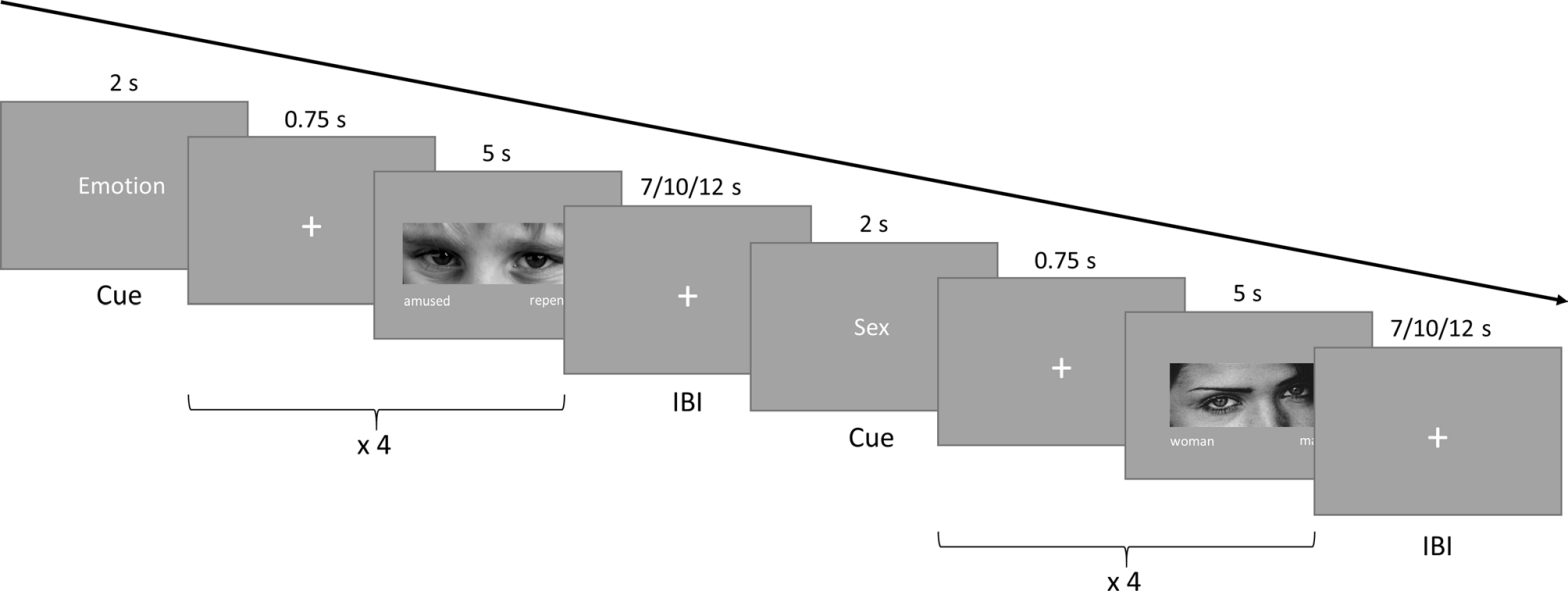
The fMRI experimental procedure was an adaptation of the RMET and NCET tasks. It consisted of four types of blocks: Adult Mind (AM; RMET adaptation), Child Mind (CM; NCET adaptation), Adult Sex (AS) and Child Sex (CS) (two control conditions). Each block consisted of a cue (Emotion/Sex), 4 photos of children or adults (depending on the task condition) and 4 fixation crosses. In each block, participants were asked to choose one of two possible terms that matched the internal state of the person in the photo (AM and CM conditions) or the sex of the person in the photo (AS and CS conditions). Blocks were presented in a pseudorandomized order and were separated with intervals. The task was divided into two sessions, and each session consisted of 18 blocks. *RMET* Reading the Mind in the Eyes Test, *NCET* Nencki Children Eyes Test, *IBI* interblock interval.

The experimental procedure was based on a previous adaptation of the *RMET* adapted for fMRI settings^6^. Participants were presented with 4 types of blocks: Adult Mind (AM,*RMET* fMRI adaptation), Child Mind (CM; *NCET* fMRI adaptation), Adult Sex (AS) and Child Sex (CS) (two control conditions). Each block was preceded by a cue indicating the type of block—an “Emotion” cue informed the participant that the next block would be one of the Mind conditions, and the “Sex” cue informed participants that it would be one of the Sex conditions. Blocks lasted for 22.25 s and consisted of 4 photos (presented for 5 s each) separated by fixation crosses (0.75 s). Blocks were separated by interblock intervals of 7, 10 or 12 s. The whole procedure took approximately 18 min and was divided into two sessions, with each session containing 18 blocks. Blocks were presented in a pseudorandomized order. Since the same pictures were presented in the Mind and the corresponding control condition, half of the participants were presented with the session in an inverse order. The experimental procedure was implemented using Presentation (ver. 20.1; Neurobehavioural Systems, Inc., Albany, CA, USA). For an overview of the procedure, see Fig. 1.

### Behavioural analysis

For the between-groups comparison of demographic data, questionnaire measures and the PENN task, we used either Student’s t-test or the Mann–Whitney U-test, depending on the distribution of the data, using R software. For the between-groups comparison of the level of education, we used the chi-squared test. Accuracy data were used to examine differences in performance between groups and task conditions. Based on participants’ performance, trials were classified as correct or incorrect (incorrect hits or misses). The aligned rank transformation was applied to accuracy data prior to ANOVA, which is the proper method for the factorial analysis of nonparametric data and accuracy data^40^. We performed ANOVA with group (2 levels: WC/NWC) as a between-subjects factor and condition (4 levels: AM/CM/AS/CS) as a within-subjects factor. To verify our behavioural hypothesis, we planned to directly compare WC and NWC in CM conditions using a one-sided Wilcoxon-Mann–Whitney U test. Additionally, we directly compared WC and NWC in other experimental conditions (AM, AS, CS) using a two-sided Wilcoxon–Mann–Whitney U test to ensure that there were no other differences between groups. As these tests were planned a priori, we did not correct for multiple comparisons^41^. Reaction time data were transformed using the Freeman–Tukey method^42^. Subsequently, ANOVA with group (2 levels: WC/NWC) as the between-subjects factor and condition (four levels: AM/CM/AS/CS) as the within-subjects factor was conducted. Reaction time data were examined to ensure that experimental conditions were more demanding than control conditions. The remaining post hoc tests were corrected using Hochberg’s correction for multiple comparisons^43^. Additionally, in the WC group, we run Pearson’s correlation between the number of years participants have worked with children (*Number of Years*), weekly hours spent in work (*Weekly Hours*) and behavioural and neuronal measures in the CM condition. All statistical analyses described in this paragraph were performed in R software^44^, with use of *emmeans*^45^ and *nlme*^46^ packages.

### MRI data acquisition

Magnetic resonance imaging data were acquired using a 3 T Siemens MAGNETOM Trio system (Siemens Medical Solutions) equipped with a 12-channel head coil. Within a single scanning session, the following images were acquired: structural localizer image, first series of functional EPI images (TR: 2,500 ms, TE: 28 ms, flip angle: 80°, voxel size: 3 × 3 × 3 mm, field of view: 216 mm, measurements: 240), second series of functional EPI images (same parameters), structural T1-weighted image (TR: 2,530 ms, TE: 3.32 ms, flip angle: 7°, voxel size: 1 × 1 × 1 mm, field of view: 256 mm), and field map (TR: 400 ms, TE: 6.81 ms, flip angle: 60°, voxel size: 3.5 × 3.5 × 3.5 mm, field of view: 216 mm).

### fMRI data preprocessing

DICOM series were converted to NIfTI using *Horos (Osirix) Bids Output Extension* (https://github.com/mslw/horos-bids-output), which is based on the dcm2niix converter (https://github.com/rordenlab/dcm2niix). Data preprocessing and analysis were performed with Statistical Parametric Mapping (SPM12, https://www.fil.ion.ucl.ac.uk/spm/). Standard preprocessing steps were used^47^, including correction for distortions related to magnetic field inhomogeneity, correction for motion by realignment to the first acquired image; coregistration of the anatomical image to the mean functional image; segmentation of the coregistered structural image with the default tissue probability maps; normalization to the MNI space; and smoothing with 6 mm FWHM Gaussian Kernel. The ARtifact Detection Tools (ART, https://www.nitrc.org/projects/artifact_detect/) software package was used to identify sources of artefacts in functional images, with a translation threshold of 2 mm and a rotation threshold of 0.02 radians.

### fMRI data analysiss

General linear modelling was used to model blood-oxygen-level dependent (BOLD) signal data for each subject at the first-level analysis. Each block was modelled with the onset of the presentation of the first photo in a given block and a duration of 22.25 s. Cues that preceded blocks were modelled with corresponding onsets and durations of 0.75 s. These predictors were convolved with a double gamma “canonical” haemodynamic response function, and a high-pass filter cut-off of 128 s was applied. Next, individual t-contrast maps were computed for each of the experimental (AM and CM) and control (AS and CS) conditions.

Initially, we conducted full factorial analysis for all participants, with age (Adult/Child) and task (Mind/Sex) as factors. The positive effect of task (Mind > Sex; *p* < 0.05, FWE corrected) was used as an explicit mask in further analysis^22^. Then, a flexible factorial design with condition (AM/CM) as the within-subjects factor and group (WC/NWC) as the between-subjects factor was performed. The interaction effect was included in the design and tested with F contrast. A voxel-wise height threshold of *p* < 0.001 (uncorrected) combined with a cluster-level extent threshold of *p* < 0.05 (FWE corrected) was applied. For post hoc analysis, we extracted mean contrast estimate values that were extracted using MarsBar (https://marsbar.sourceforge.net/index.html) from ROIs defined by the clusters with significant activation obtained in the interaction F contrast. The extracted values were compared using the emmeans package in R^44,45^ and were corrected using Hochberg’s method^43^. All brain areas reported in the study are labelled according to the automated anatomical labelling (AAL2)^48,49^ atlas applied in bspmview (https://www.bobspunt.com/bspmview). Additionally, we used the Neurosynth (https://www.neurosynth.org) website to evaluate whether the coordinates for significant activations in the flexible factorial design corresponded to functional maps reported in the literature.

## Results

### Behavioural results

#### Control tests

We did not observe any between-groups differences in any of the control measures. The results are summarized in Table 1.

**Table 1:** Group comparisons of behavioural and self-reported measures.

	Group		*p* value
	WC	NWC	
	Mean (SD)		
**T-test**			
Years of education	15.9 (3.5)	15.9 (2.26)	0.938
Age	24.6 (3.3)	23.2 (2.5)	0.132
TRS-S	23.7 (4.25)	23.7 (4.1)	1
Empathic concern (IRI)	36.0 (3.99)	34.7 (3.68)	0.244
Personal distress (IRI)	24.0 (4.28)	25.1 (5.85)	0.616
Perspective taking (IRI)	31.9 (3.38)	31.3 (1.73)	0.674

	WC	NWC	*p* value
	Median (IQR)		
**Mann–Whitney U test**			
PENN ER-40	35 (3.25)	34 (3)	0.228
Hinting task score	17 (7)	16.5 (15)	0.972
Hinting task hints	12 (7)	11.5 (8)	0.958

	*p* value		
**Chi-squared test**			
Education	0.528		

*WC* working with children, *NWC* not working witch children.

#### RMET and NCET

**Figure 2:**
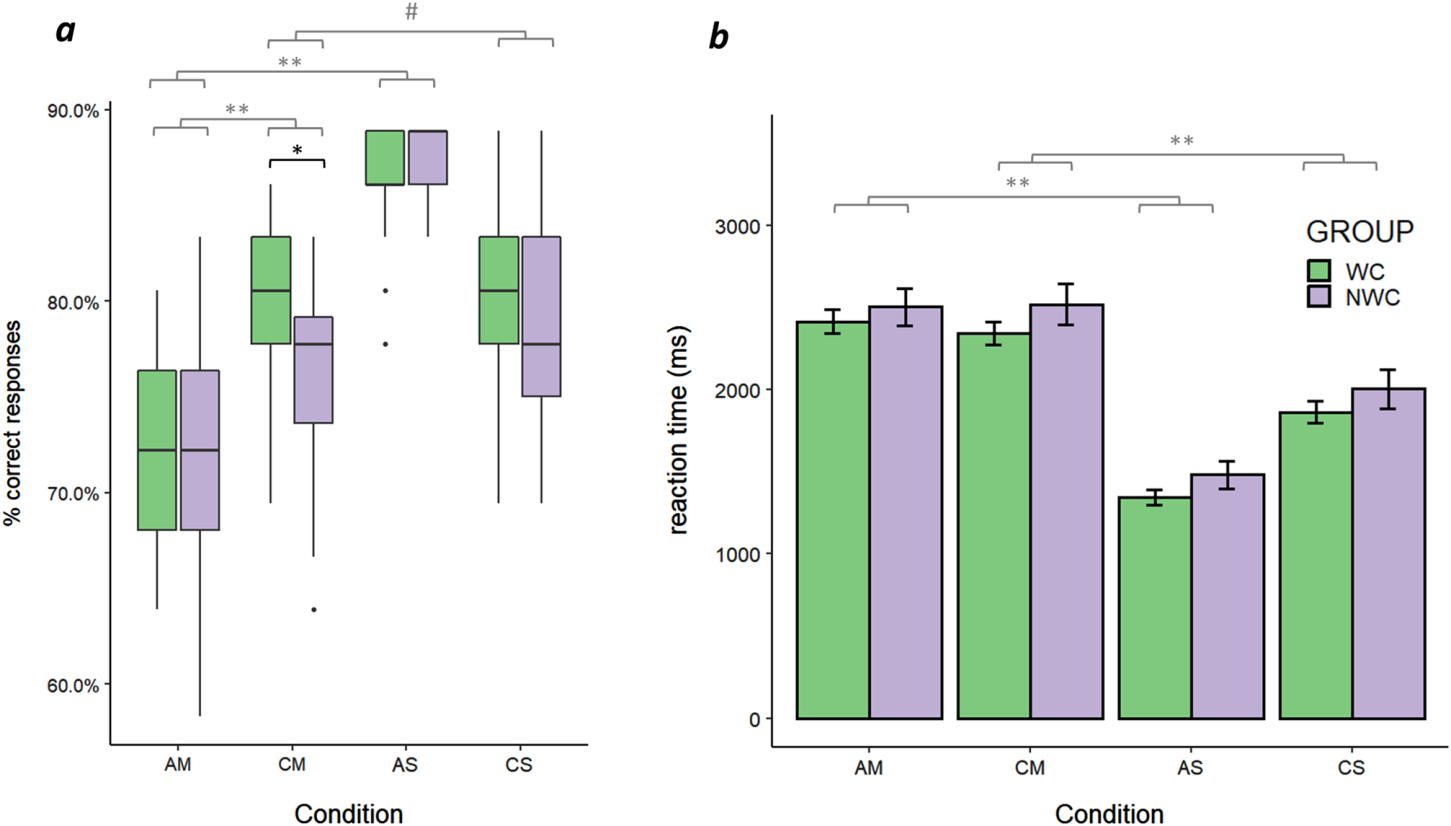
Behavioural results of RMET and NCET. (**a**) Accuracy in different task conditions for the two groups. Significant post hoc tests of the main effect of condition are marked in grey. A significant post hoc test of the interaction between group and condition is marked in black. (**b**) The mean reaction times in different task conditions for the two groups. Error bars represent SEs. Significant post hoc tests of the main effect of condition are marked in grey. Groups: *WC* working with children, *NWC* not working with children; Conditions: *AM* adult mind, *CM* child mind, *AS* adult sex, *CS* child sex; #*p* < 0.1; *p < 0.05; **p < 0.001.

An analysis of accuracy revealed a significant effect of condition (F(3,108) = 69.18, *p* < 0.001, η2 = 0.54) but no effect of group (F(1,36) = 0.94, *p* = 0.34, η2 = 0.01). There was a trend towards a significant effect of the interaction between group and condition (F(3,108) = 2.65, *p* = 0.052, η2 = 0.04). Post hoc tests showed that AM was more difficult than AS (T = − 14.18, *p* < 0.001), AM was more difficult than CM (T = − 4.9, *p* < 0.001), and AS was less difficult than CS (T = 7.5, *p* < 0.001). There was also a trend showing that CM was more difficult than CS (T = − 1.8, *p* = 0.075). Post hoc tests of the interaction effect showed that WC scored higher than NWC in CM (U = 255, *p* = 0.028, r effect size = 0.18), whereas there were no significant differences between groups in AM (U = 187.5, *p* = 0.85), AS (U = 137, *p* = 0.13) or CS (U = 210, *p* = 0.4) conditions (Fig. 2a).

An analysis of response times revealed a significant effect of condition (F(3,108) = 433.7, *p* < 0.001, η2 = 0.57) but no significant effect of group (F(1,36) = 1.02, *p* = 0.32, η2 = 0.03) or an interaction between group and condition (F(3,108) = 0.55, *p* = 0.65, η2 = 0.001). Post hoc tests showed that reaction time was higher in AM than in AS (T = 27.9, *p* < 0.001), in CM than in CS (T = 13.3, *p* < 0.001) and in AS than in CS (T = − 13.9, *p* < 0.001) conditions. There was no difference in response times between AM and CM conditions (T = 0.71, *p* = 0.47; Fig. 2b).

### fMRI results

**Figure 3:**
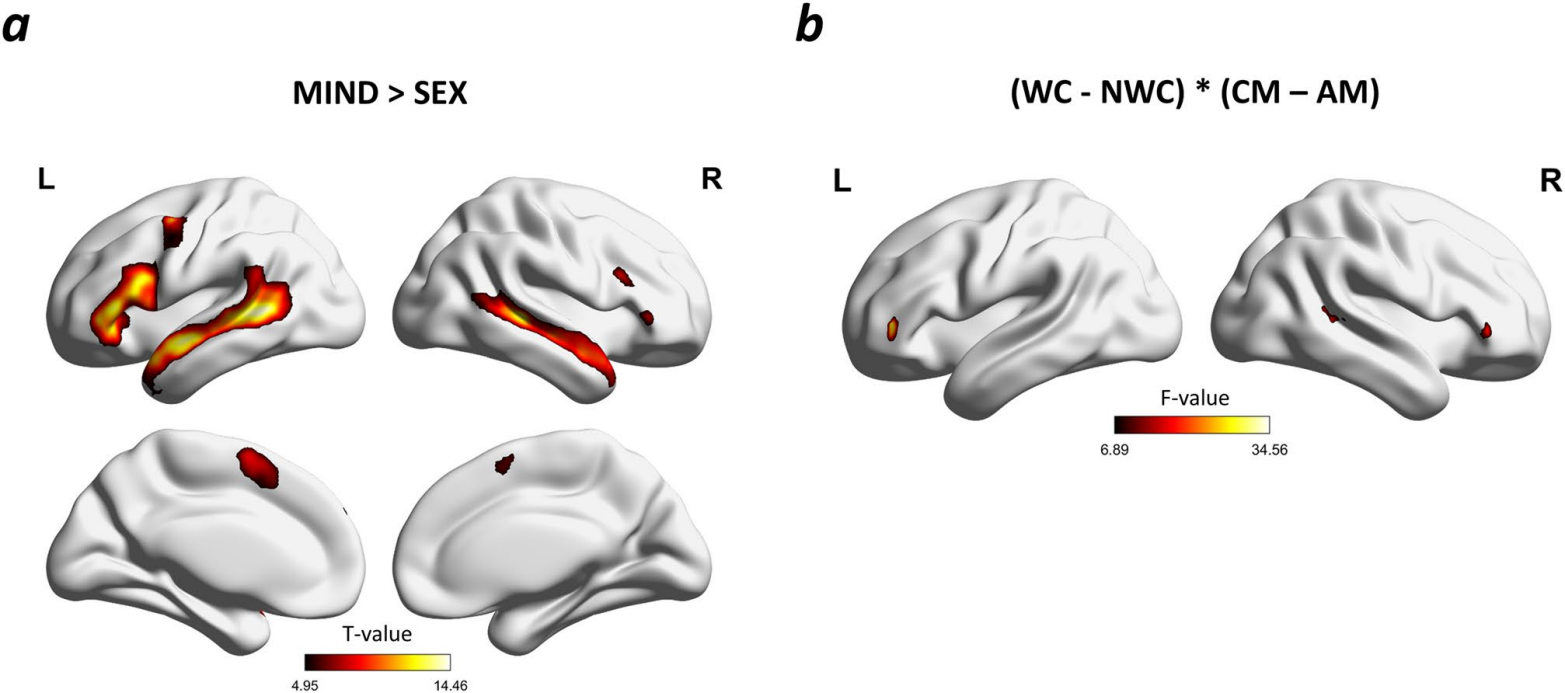
(**a**) Whole-brain statistical parametric maps representing brain activation during the attribution of the mental states of others (MIND > SEX); corrected for multiple comparisons (FWE; *p* < 0.05). (**b**) Whole-brain statistical parametric maps representing the F contrast interaction between group and condition (WC > NWC) * (CM > AM) with a voxel-wise height threshold of *p* < 0.001 (uncorrected) combined with a cluster-level extent threshold of *p* < 0.05 (corrected for multiple comparisons using the FWE. Analysis was limited to volumes from a. Groups: *WC* working with children, *NWC* not working with children, Conditions: *AM* adult mind, *CM* child mind, *MIND* AM and CM, *SEX* AS and CS.

Whole brain analysis for all participants revealed that the attribution of mental states to others (AM and CM > AS and CS) activated a broad network consisting of activation surrounding the bilateral STS and superior temporal gyrus (STG), bilateral inferior frontal gyri (IFG), bilateral middle temporal gyrus (MTG), right temporal pole (TP) and left middle frontal gyrus (MFG) (Fig. 3a). A more thorough description of the clusters and peaks obtained in the analysis is presented in Table 2. The F contrast of the interaction between group and condition (WC − NWC) * (CM − AM) resulted in significant clusters of voxels in the bilateral IFG and right pSTS (Table 3; Fig. 3b). The results from F contrast interaction were then explored using post hoc tests on the estimated mean values of contrasts extracted from left IFG, right IFG and right pSTS.

**Table 2:** Peak level activations related to understanding the minds of others compared to sex recognition.

Contrast	Region label	Hemisphere	Cluster extent	t-value	MNI coordinates			p FWE
					x	y	z	
AM and CM > AS and CS	Inferior frontal gyrus, pars orbitalis	L	7,951	15.731	− 48	14	22	< 0.001
	Middle temporal gyrus	L		14.424	− 58	− 48	6	< 0.001
	Superior temporal gyrus	L		14.419	− 54	− 6	− 10	< 0.001
	Middle temporal gyrus	R	2,451	13.264	52	− 34	2	< 0.001
	Middle temporal gyrus	R		10.896	54	− 2	− 16	< 0.001
	Temporal pole: superior temporal gyrus	R		10.150	48	16	− 24	< 0.001
	Supplementary motor area	L/R	686	10.819	− 4	10	54	< 0.001
	Inferior frontal gyrus, pars triangularis	R	632	10.279	52	32	0	< 0.001
	Inferior frontal gyrus, pars triangularis	R		8.889	46	18	24	< 0.001
	Cerebellum	R	901	8.949	18	− 68	− 26	< 0.001
	Cerebellum crus	R		7.401	40	− 66	− 26	< 0.001
	Cerebellum	L		5.527	− 8	− 76	− 18	< 0.001
	Inferior occipital gyrus	R	142	6.669	− 22	− 98	− 8	< 0.001
	Superior frontal gyrus, medial	L	44	6.525	− 10	54	28	< 0.001
	Precentral gyrus	R	14	6.270	56	2	44	< 0.001
	Superior temporal gyrus	R	13	6.075	66	− 38	22	0.001
	Calcarine fissure and surrounding cortex	L	74	5.987	− 14	− 72	10	0.001
	Cerebellum crus	L	10	5.964	− 16	− 72	− 28	0.001
	Vermis	R	42	5.840	0	− 52	− 34	0.002
	Thalamus	L	34	5.832	− 8	− 16	8	0.002
	Lenticular nucleus, Putamen	R	31	5.540	20	14	2	0.006
	Middle frontal gyrus	R	13	5.522	46	2	56	0.006
	Precentral gyrus	R	25	5.208	40	− 4	46	0.020

The table shows all the local maxima separated by more than 8 mm. Regions were automatically labelled using automatic anatomical labelling. x, y, and z of the Montreal Neurological Institute (MNI) coordinates in the left–right, anterior–posterior, and inferior-superior dimensions, respectively.

**Table 3:** Peak level activations related to understanding the minds of others compared to sex recognition.

Contrast	Associations neurosynth	Region label (AAL2)	Hemisphere	Cluster extent	F-value	MNI coordinates			p FWE
						x	y	z	
(WC − NWC) * (CM − AM)	Inferior frontal gyrus	Inferior frontal gyrus, pars triangularis	L	81	34.215	− 42	40	4	0.009
	Ventrolateral prefrontal	Inferior frontal gyrus, pars triangularis	R	50	20.560	50	34	− 2	0.240
	Posterior superior temporal sulcus	Superior temporal gyrus	R	55	18.450	54	− 34	8	0.387

A voxel-wise height threshold of *p* < 0.001 (uncorrected) combined with a cluster-level extent threshold of *p* < 0.05 (corrected for multiple comparisons using the FWE rate) was applied. Activations surviving a peak-level FWE-corrected threshold of *p* < 0.05 are marked with bold text. The table shows all the local maxima separated by more than 8 mm. Regions were automatically labelled using automatic anatomical labelling. x, y, and z of the Montreal Neurological Institute (MNI) coordinates in the left–right, anterior–posterior, and inferior–superior dimensions, respectively.

#### Left IFG

**Figure 4:**
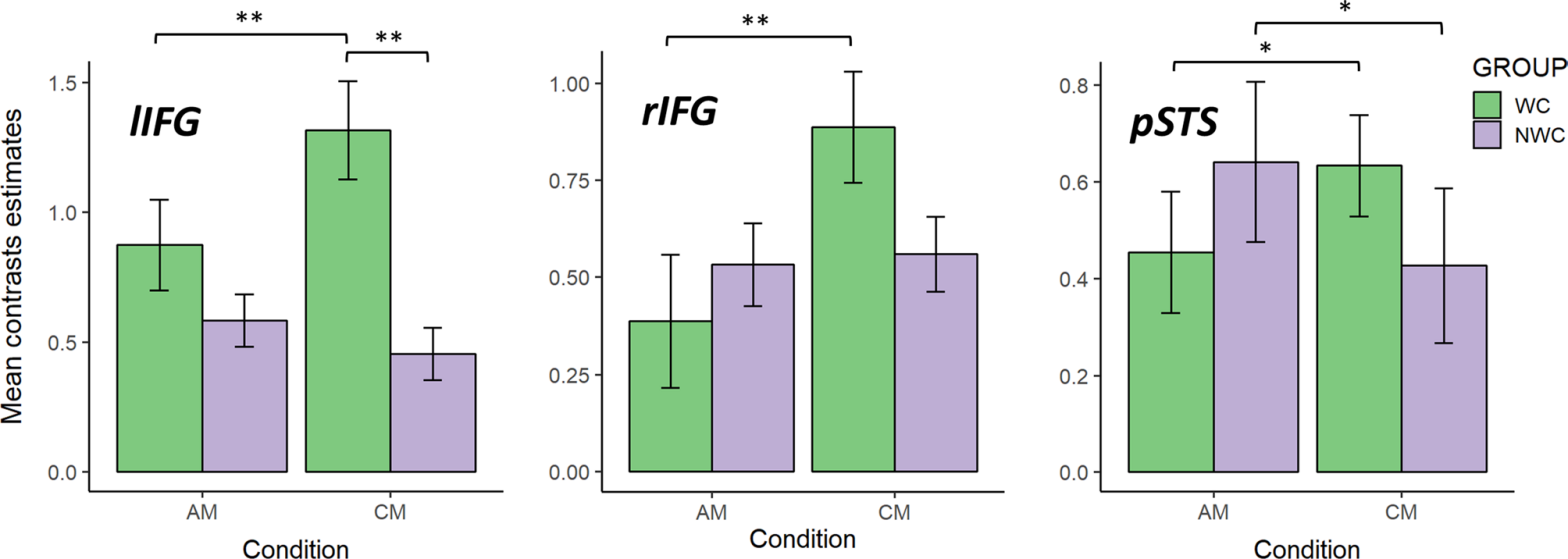
The mean contrast estimates for the two experimental conditions for the two groups; Error bars represent SEs. Significant post hoc tests of the interaction between group and condition are marked with black brackets and symbols. Hochberg’s correction for multiple comparisons was applied. Groups: *WC* working with children, *NWC* not working with children; Conditions: *AM* adult mind, *CM* child mind; Regions: *lIFG* left inferior frontal gyrus, *rIFG* right inferior frontal gyrus, *pSTS* right posterior superior temporal sulcus. *p < 0.05; **p < 0.001.

Post hoc comparison revealed that in the CM condition, the WC group had stronger activation than the NWC group (t = 4.13; *p* < 0.001) and that the WC group had stronger activation in the CM condition than the AM condition (t = 4.59; *p* < 0.001) (Fig. 4).

#### Right IFG

Post hoc comparisons revealed that the WC group had stronger activation in the CM condition than the AM condition (t = 4.26; *p* < 0.001; Fig. 4).

#### Right pSTS

Post hoc comparisons revealed that the WC group had stronger activation in the CM condition than the AM condition (t = 2.53; *p* = 0.048). The opposite pattern was observed in the NWC group, which had stronger activation in AM condition than the CM condition (t = 3.01; *p* = 0.019) (Fig. 4).

### Correlations between time spend with children, behavioural and neuronal measures

We found that reaction times in CM were negatively correlated with *Number of Years* (r = − 0.53; *p* = 0.024). There were no other significant correlations (Table 4.)

**Table 4:** Pearson’s R correlations between measures of time spend with children, behavioural and neuronal measures in CM condition, in WC group.

	CM reaction times	CM accuracy	CM L IFG	CM R IFG	CM pSTS
Number of years (*n* = 18)	− 0.53*	0.21	− 0.13	− 0.25	− 0.40
Weekly hours (*n* = 16)	0.18	0.05	0.32	− 0.07	− 0.16

CM—Child mind; WC—working with children; Number of years—number of years participants have been working with children; Weekly hours—weekly hours spend in work. *p < 0.05.

## Discussion

The phenomena of being able to better remember the faces of members of our own age and own race groups have been well documented^20–27^. These phenomena are described as own-age bias and own-race bias, respectively. Currently, there is also evidence for own-race bias in understanding mental states in the RMET. This bias can be reduced by gaining experience with other ethnic groups. However, it is unclear whether a similar effect of experience occurs in adults who work with children, reducing own-age bias. Clarifying this topic can improve our understanding of how experience affects TOM and underlying neuronal processes.

To answer this question, we recruited two groups of adults who were either working with or not working with children and asked them to perform the NCET (CM condition) and RMET (AM condition) tasks in an fMRI setting. We showed that the WC group scored better in the CM than NWC, while there were no between-group differences in the AM condition. When comparing MIND (AM and CM) to SEX (AS and CS) conditions, we observed substantial activation in the bilateral IFG, temporal poles and STS, regions that had previously been reported in studies using the RMET. Additionally, we found an effect of the interaction ((NWC–WC) * (CM–AM)) in the bilateral IFG and right pSTS. Specifically, in the left IFG, in the CM condition, the WC group had stronger activation than NWC, and the WC group had stronger activation in the CM condition than in the AM condition. A similar difference between the CM and AM conditions was observed for the WC group, in the right IFG and right pSTS. Additionally, in the right pSTS, NWC were characterized by stronger activation during the AM condition than the CM condition.

### Behavioural differences

We found that the WC group performed better than the NWC group in the CM condition, as we hypothesized. At the same time, there were no differences between groups in other experimental conditions. This result is in line with previous studies on an increased ability to remember children’s faces in adults who work with children. A similar improvement was observed in people who live outside their culture of origin. Anatolian Dutch and Moroccan Dutch individuals did not differ in their performance of their own-culture RMET and the Caucasian RMET, while Caucasian Dutch individuals performed worse on the other cultures’ RMETs^9^. The authors of this study suggested that bicultural individuals need to adjust to the Dutch (majority) culture in situations such as work or school, while during interactions with their relatives, they still need to act according to their primary culture. Another study that provided evidence for experience-based improvement in TOM abilities involved Asians living in Canada^8^. Although these subjects performed worse in the Caucasian RMET than in the East Asian RMET, their accuracy in the Caucasian RMET increased as a function of the time they had lived in Canada, their experience interacting with Caucasians, how positive their view on Canadian values was, and how much their identification with their primary culture had decreased.

We did not find behavioural effects of own-age bias. For all participants, the AM condition was harder than CM. This might have been caused by the fact that children’s facial expressions are more straightforward than the facial expressions of adults. Basic emotions such as sadness, anger and happiness were more easily recognized if they were expressed by children than adults^50^. However, disgust was the only basic emotion that was better recognized if presented by adults. Additionally, in our study, children’s facial expressions might have been easier to correctly match with a given adjective, thus resulting in a lack of behavioural effects related to the own-age bias.

Another explanation of such results would be the difference between valence and/or intensity of the stimuli in NCET and RMET. Unfortunately, following the procedure of Baron-Cohen et al.^18^ we did not collect the valence and arousal ratings of the stimuli, thus we cannot conclude about the possible impact of these factors. Since we did not observe an effect of the own-age bias, it is more appropriate to ascribe the increased ability to recognize children mental states, observed in the WC group, as caused by familiarity or experience with children. This is further strengthened by the fact that the number of years the participants in the WC group had worked with children was inversely related to the reaction time in the CM condition. Familiarity was described as a potential cause of a reduction of the own-age bias in face recognition in adults who work with children^16,17^. It was also shown to improve various cognitive skills^51,52^, in particular recognition of face stimuli^53,54^.

### Differences in pSTS activity

In the NWC group, pSTS was activated more in the AM condition than CM. The opposite was observed in the WC group, in which the pSTS was more active in the CM condition than the AM condition. The pattern of activation in the NWC group resembles results reported in studies of own-race bias in the RMET^7^. All participants in this study were characterized by lower activity for other races than for the own-race RMET. This lower pSTS activation to the other-race RMET was also associated with the effect of own-race bias (better performance in the own-race RMET). However, based on the neuronal activation in the NWC group we cannot conclude the occurrence of own-age bias as all participants performed better in the CM condition. The pSTS is a core region in the network responsible for social information processing, serving as a hub communicating with many other regions^24^. This region receives input from sensory regions and is sensitive to social information. The pSTS was shown to be activated specifically when socially relevant stimuli were contrasted with irrelevant stimuli^29^. Information about social cues is sent to the IFG and the inferior parietal lobule (IPL), which are responsible for understanding others’ actions and emotions^25,27^ by referring them to our own. Next, the signal is sent back to the pSTS where it can be transferred further for more advanced TOM processing, such as belief attribution, based on prior information. The increased activation of the pSTS when understanding the mental states of children might reflect the increased importance of such interactions in the WC group. The increased importance of these interactions was previously proposed as being responsible for the ability of teachers to better remember children’s faces^17^ and for better performance in RMETs of other cultures^9^. The other explanation would simply be a better ability to process sensory information derived from the eye region, in other words, sensory expertise caused by familiarity. The pSTS is also engaged in face-selective processing and activates stronger to familiar vs unfamiliar faces^55,56^. In a recent study, the pSTS was found to be related to person-selective processing, irrespectively of modality^57^. Therefore the increased activation of pSTS during recognizing the mental states of children in the WC group might reflect an increased familiarity with children. Last, this effect might have been caused by increased activity in the mirror neuron system, corresponding to increased empathy with children. This explanation is highly plausible, as we also observed between- and within-groups differences in the bilateral IFG.

### Differences in IFG activity and the mirror neuron system

For the WC group, the activation in the bilateral IFG was higher in the CM condition than in the AM condition, similar to what we observed in the pSTS. Additionally, the WC group was characterized by increased activity in the left IFG compared to NWC in the CM condition.

IFG and IPL are parts of the Human Mirror Neuron System (MNS), a group of neurons activated by motor performance as well as observing movements performed by others^58^. MNS was also linked to action understanding, imitation^59^, understanding intentions^60^ and also emotions of others, thanks to facial mimicry^61,62^. According to the simulation theory, MNS is also the basis for TOM and allows the observer to simulate a mental state that corresponds to the state of the observed person^63^. The activation of the IFG is typically reported in studies using RMET-type tasks^23^, and it is crucial to correctly perform the RMET. Patients with brain lesions in the IFG have been shown to have decreased accuracy in the RMET^26^. Transcranial magnetic stimulation of the IFG was shown to increase reaction times during the RMET and disrupt EEG rhythms related to mirror neurons activity^64^. The RMET requires emotional and semantic processing. Similarly, IFG function is believed to be related to facial mimicry^27,65,66^ and storing semantic representations of others’ mental states^26^. However, increased activity in the IFG of those in the WC group when understanding the mental states of children is unlikely to be related to differences in purely semantic processing, as both groups did not differ in their vocabulary knowledge, and the TRS-S score was not related to AM or CM accuracy. Moreover, the AM condition also required the semantic processing of similar adjectives, but no differences in the activation of the IFG during the AM condition were observed. It is more plausible that the WC group expressed increased facial mimicry and had better ability to simulate children’s mental states when viewing children’s photographs, which resulted in a more accurate choice of descriptions of mental states in the CM condition. Interestingly, increased activation of left IFG was also observed in a group of older adults while they performed RMET (comprised mostly of photographs of young adults) in fMRI^67^. Elderly subjects did not differ from young adults, in accuracy, thus the increased engagement of IFG might have been needed to better understand the mental states of members of different age-group, similar to what was observed in the WC group in our study.

An increase in the activation of brain regions related to mirroring and theory of mind has been previously reported by different groups of experts in specific fields^68,69^. For example, when watching archery videos, a group of expert archers showed stronger activation in the IPL, pSTS and inferior prefrontal cortex than a non-archer control group. This increased activation was interpreted as an increased number of representations in the human mirror neuron system. Similarly, in our study, the WC group could have shown an increase in the number of representations of children’s facial expressions and/or mental states. Increased activity in bilateral IFG in the WC group supports the role of MNS in the ability to decode the mental states.

### Study implications

Our study is the first to focus on specific expertise in understanding mental states, so these results need to be treated with caution and further explored. Further studies could determine whether this effect could be generalized to other age-groups like adolescents or the elderly. Nevertheless, training-induced neuroplasticity changes in regions related to TOM processing have already been reported in the literature^70^. Our results have implications for childhood education. It shows the potential of personal experience in improving the ability to understand the mental states of children. One may ask to what extent such personal experience in the form of a practical internship (or even having own children) can influence the ability to understand the mental states of children compared to formal pedagogical education. Additionally, our study may shed light on the contact hypothesis which is the idea that interpersonal contact can improve intergroup relations and can effectively reduce prejudice between various social groups (Allport, 1954). Although this hypothesis found support in hundreds of studies (Pettigrew and Tropp, 2006), the psychological processes involved in this improvement are still debated in the literature. One may speculate that one such mediating mechanism is TOM. The prolonged intergroup contact may facilitate the ability to understand mental states of other groups’ members which in turn helps to take the perspective of those members and to empathize with them.

### Study limitations and future directions

We used experimental tasks that measure mental state decoding and can engage both affective and cognitive TOM. Substantial step forward would be to investigate whether familiarity with children affects mental state reasoning and use tasks which target affective and cognitive components specifically. Additionally, we do not know whether increased contact with children is the reason for better accuracy in CM, in the WC group or whether people who are better at thinking about the minds of children are more likely to work with them. Currently, we know that a similar effect of experience on the accuracy of out-group RMET performance is observed in people who live in multicultural societies outside their culture of origin. Future studies should explore the underlying neuronal mechanism of these behavioural results and compare them to the results obtained in our study. Another substantial step forward would be to investigate whether own-age, as well as other in-group biases, affect affective and cognitive TOM using experimental tasks targeting those processes more specifically. Lastly, since behavioural and neuronal differences in RMET, were observed between children, adolescent and adults^22^ investigating those groups with NCET might expand our understanding of TOM development.

## Conclusions

In summary, we showed that familiarity with children improved the ability to understand the mental states of children in the WC group. In line with the behavioural results, we observed increased activation in the right pSTS and bilateral IFG during the attribution of mental states to children. This was not observed in the NWC group, in which the pSTS was more active during recognizing mental states of adults. Therefore, the engagement of these regions is required to improve the mindreading from the eye region. These differences in the brain’s activity provide novel information about how experience with out-groups can shape behaviour and neuronal processing related to TOM.

## Supplementary information

Supplementary information

## Data Availability

Behavioural data, 1st level contrasts for individual subjects and 2nd level thresholded statistical maps are available to download from https://osf.io/47hdc/.

## References

[1] Baron-Cohen S, Golan O, Ashwin E (2009). Can emotion recognition be taught to children with autism spectrum conditions?. Philos. Trans. R. Soc. B Biol. Sci..

[2] Frith CD, Frith U (2006). The neural basis of mentalizing. Neuron.

[3] Premack D, Woodruff G (1978). Does the chimpanzee have a theory of mind?. Behav. Brain Sci..

[4] Tager-Flusberg H (2000). A componential view of theory of mind: Evidence from Williams syndrome. Cognition.

[5] Sabbagh MA (2004). Understanding orbitofrontal contributions to theory-of-mind reasoning: Implications for autism. Brain Cogn..

[6] Shamay-Tsoory SG, Harari H, Aharon-Peretz J, Levkovitz Y (2010). The role of the orbitofrontal cortex in affective theory of mind deficits in criminal offenders with psychopathic tendencies. Cortex.

[7] Adams RB (2010). Cross-cultural Reading the Mind in the Eyes: An fMRI investigation. J. Cogn. Neurosci..

[8] Bjornsdottir RT, Rule NO (2016). On the relationship between acculturation and intercultural understanding: Insight from the Reading the Mind in the Eyes test. Int. J. Intercult. Relat..

[9] van der Meulen A, de Ruyter D, Blokland A, Krabbendam L (2019). Cross-cultural mental state reading ability in Antillean Dutch, Moroccan Dutch, and Dutch young adults. J. Cross. Cult. Psychol..

[10] Meissner CA, Brigham JC (2001). Thirty years of investigating the own-race bias in memory for faces: A meta-analytic review. Psychol. Public Policy Law.

[11] Ebner NC, He Y, Johnson MK (2011). Age and emotion affect how we look at a face: Visual scan patterns differ for own-age versus other-age emotional faces. Cogn. Emot..

[12] Ebner NC, Johnson MK (2010). Age-group differences in interference from young and older emotional faces. Cogn. Emot..

[13] He Y, Ebner NC, Johnson MK (2011). What predicts the own-age bias in face recognition memory?. Soc. Cogn..

[14] Anastasi JS, Rhodes MG (2005). An own-age bias in face recognition for children and older adults. Psychon. Bull. Rev..

[15] Rhodes MG, Anastasi JS (2012). The own-age bias in face recognition: A meta-analytic and theoretical review. Psychol. Bull..

[16] Cassia VMC, Picozzi M, Kuefner D, Casati M (2009). Why mix-ups don’t happen in the nursery: Evidence for an experience-based interpretation of the other-age effect. Q. J. Exp. Psychol..

[17] Harrison V, Hole GJ (2009). Evidence for a contact-based explanation of the own-age bias in face recognition. Psychon. Bull. Rev..

[18] Baron-Cohen S, Wheelwright S, Hill J, Raste Y, Plumb I (2001). The ‘Reading the Mind in the Eyes’ test revised version: A study with normal adults, and adults with Asperger syndrome or high-functioning autism. J. Child Psychol. Psychiatry.

[19] Mitchell RLC, Phillips LH (2015). The overlapping relationship between emotion perception and theory of mind. Neuropsychologia.

[20] Adolphs R, Baron-Cohen S, Tranel D (2002). Impaired recognition of social emotions following amygdala damage. J. Cogn. Neurosci..

[21] Meinhardt-Injac B, Daum MM, Meinhardt G, Persike M (2018). The two-systems account of theory of mind: Testing the links to social-perceptual and cognitive abilities. Front. Hum. Neurosci..

[22] Moor GB (2012). Neurodevelopmental changes of reading the mind in the eyes. Soc. Cogn. Affect. Neurosci..

[23] Schurz M, Radua J, Aichhorn M, Richlan F, Perner J (2014). Fractionating theory of mind: A meta-analysis of functional brain imaging studies. Neurosci. Biobehav. Rev..

[24] Yang DY-J, Rosenblau G, Keifer C, Pelphrey KA (2015). An integrative neural model of social perception, action observation, and theory of mind. Neurosci. Biobehav. Rev..

[25] Keysers C, Gazzola V (2010). Social neuroscience: Mirror neurons recorded in humans. Curr. Biol..

[26] Dal Monte O (2014). The left inferior frontal gyrus is crucial for reading the mind in the eyes: Brain lesion evidence. Cortex.

[27] Rymarczyk K, Żurawski Ł, Jankowiak-Siuda K, Szatkowska I (2018). Neural correlates of facial mimicry: Simultaneous measurements of EMG and BOLD responses during perception of dynamic compared to static facial expressions. Front. Psychol..

[28] Uono S (2017). Neural substrates of the ability to recognize facial expressions: A voxel-based morphometry study. Soc. Cogn. Affect. Neurosci..

[29] Bahnemann M, Dziobek I, Prehn K, Wolf I, Heekeren HR (2010). Sociotopy in the temporoparietal cortex: Common versus distinct processes. Soc. Cogn. Affect. Neurosci..

[30] Schobert A-K, Corradi-Dell’Acqua C, Frühholz S, van der Zwaag W, Vuilleumier P (2018). Functional organization of face processing in the human superior temporal sulcus: A 7T high-resolution fMRI study. Soc. Cogn. Affect. Neurosci..

[31] Marchewka A, Żurawski Ł, Jednoróg K, Grabowska A (2014). The Nencki Affective Picture System (NAPS): Introduction to a novel, standardized, wide-range, high-quality, realistic picture database. Behav. Res. Methods.

[32] Olderbak S (2015). A psychometric analysis of the reading the mind in the eyes test: Toward a brief form for research and applied settings. Front. Psychol..

[33] Peterson E, Miller SF (2012). The eyes test as a measure of individual differences: How much of the variance reflects verbal IQ?. Front. Psychol..

[34] Gur RC (2002). A method for obtaining 3-dimensional facial expressions and its standardization for use in neurocognitive studies. J. Neurosci. Methods.

[35] Corcoran R, Mercer G, Frith CD (1995). Schizophrenia, symptomatology and social inference: Investigating “theory of mind” in people with schizophrenia. Schizophr. Res..

[36] Krawczyk, M., Schudy, A., Jarkiewicz, M. & Okruszek, Ł. Polish version of The Hinting Task—Pilot study with patients with schizophrenia. *Psychiatr. Pol.* 10.12740/PP/112265 33386724

[37] Matczak, A., Jaworksa, A. & Martowska, K. *Test Rozumienia Słów – Wersja Standard i Wersja dla Zaawansowanych*. (Pracownia Testów Psychologicznych Polskeigo Towarzystwa Psychologicznego, 2012).

[38] Davis MH (1980). A multidimensional approach to individual differences in empathy. J. Pers. Soc. Psychol..

[39] Kaźmierczak M, Plopa M, Retowski S (2007). Skala Wrażliwości Empatycznej. Przegląd Psychol..

[40] Wobbrock, J. O., Findlater, L., Gergle, D. & Higgins, J. J. The aligned rank transform for nonparametric factorial analyses using only anova procedures. In *Proceedings of the 2011 Annual Conference on Human Factors in Computing Systems—CHI ’11* 143 (ACM Press, 2011). 10.1145/1978942.1978963.

[41] Keppel G, Wickens TD (2004). Design and Analysis: A Reseacher’s Handbook.

[42] Freeman MF, Tukey JW (1950). Transformations related to the angular and the square root. Ann. Math. Stat..

[43] Hochberg Y (1988). A sharper Bonferroni procedure for multiple tests of significance. Biometrika.

[44] R Core Team. *R: A Language and Environment for Statistical Computing* (R Foundation for Statistical Computing, Vienna, 2018). https://www.R-project.org/.

[45] Lenth, R. emmeans: Estimated Marginal Means, aka Least-Squares Means. R package version 1.4.1. (2019).

[46] Pinheiro, J., Bates, D., DebRoy, S., Sarkar, D. & R Core Team. nlme: Linear and Nonlinear Mixed Effects Models. R package version 3.1-140 (2019).

[47] Poldrack RA, Nichols T, Mumford J (2011). Handbook of Functional MRI Data Analysis. Handbook of Functional MRI Data Analysis.

[48] Rolls ET, Joliot M, Tzourio-Mazoyer N (2015). Implementation of a new parcellation of the orbitofrontal cortex in the automated anatomical labeling atlas. Neuroimage.

[49] Tzourio-Mazoyer N (2002). Automated anatomical labeling of activations in SPM using a macroscopic anatomical parcellation of the MNI MRI single-subject brain. Neuroimage.

[50] Griffiths S, Penton-Voak IS, Jarrold C, Munafò MR (2015). No own-age advantage in children’s recognition of emotion on prototypical faces of different ages. PLoS ONE.

[51] Malinowski P, Hübner R (2001). The effect of familiarity on visual-search performance: Evidence for learned basic features. Percept. Psychophys..

[52] Xie W, Zhang W (2017). Familiarity increases the number of remembered Pokémon in visual short-term memory. Mem. Cognit..

[53] Balas B, Cox D, Conwell E (2007). The effect of real-world personal familiarity on the speed of face information processing. PLoS ONE.

[54] Jackson MC, Raymond JE (2006). The role of attention and familiarity in face identification. Percept. Psychophys..

[55] Bobes MA, Lage Castellanos A, Quiñones I, García L, Valdes-Sosa M (2013). Timing and tuning for familiarity of cortical responses to faces. PLoS ONE.

[56] Gobbini MI, Haxby JV (2007). Neural systems for recognition of familiar faces. Neuropsychologia.

[57] Tsantani M, Kriegeskorte N, McGettigan C, Garrido L (2019). Faces and voices in the brain: A modality-general person-identity representation in superior temporal sulcus. Neuroimage.

[58] Rizzolatti G, Sinigaglia C (2010). The functional role of the parieto-frontal mirror circuit: Interpretations and misinterpretations. Nat. Rev. Neurosci..

[59] Rizzolatti G, Craighero L (2004). The mirror-neuron system. Annu. Rev. Neurosci..

[60] Iacoboni M (2005). Grasping the intentions of others with one’s own mirror neuron system. PLoS Biol..

[61] Schulte-Rüther M, Markowitsch HJ, Fink GR, Piefke M (2007). Mirror neuron and theory of mind mechanisms involved in face-to-face interactions: A functional magnetic resonance imaging approach to empathy. J. Cogn. Neurosci..

[62] Rymarczyk K, Żurawski Ł, Jankowiak-Siuda K, Szatkowska I (2019). Empathy in facial mimicry of fear and disgust: Simultaneous EMG-fMRI recordings during observation of static and dynamic facial expressions. Front. Psychol..

[63] Gallese V, Goldman A (1998). Mirror neurons and the simulation theory of mind-reading. Trends Cogn. Sci..

[64] Keuken MC (2011). The role of the left inferior frontal gyrus in social perception: An rTMS study. Brain Res..

[65] Lee TW, Josephs O, Dolan RJ, Critchley HD (2006). Imitating expressions: Emotion-specific neural substrates in facial mimicry. Soc. Cogn. Affect. Neurosci..

[66] Jabbi M, Keysers C (2008). Inferior frontal gyrus activity triggers anterior insula response to emotional facial expressions. Emotion.

[67] Castelli I (2010). Effects of aging on mindreading ability through the eyes: An fMRI study. Neuropsychologia.

[68] Abreu AM (2012). Action anticipation beyond the action observation network: A functional magnetic resonance imaging study in expert basketball players. Eur. J. Neurosci..

[69] Kim Y-T (2011). Neural correlates related to action observation in expert archers. Behav. Brain Res..

[70] Valk SL (2017). Structural plasticity of the social brain: Differential change after socio-affective and cognitive mental training. Sci. Adv..

