# Supplementary material for: Familiarity with children improves the ability to recognize children’s mental states: an fMRI study using the Reading the Mind in the Eyes Task and the Nencki Children Eyes Test
Source: Sci Rep. 2020 Jul 31;10:12964. doi: 10.1038/s41598-020-69938-4 (PMC7395771; doi:10.1038/s41598-020-69938-4)
Supplement: Supplementary file 1 — Supplementary information [file 41598_2020_69938_MOESM1_ESM.docx]

**Familiarity with children improves the ability to recognize children’s mental states - an fMRI study using RMET and NCET**

Jan Szczypiński^1,2*^, Anna Alińska^1^, Marek Waligóra^1,3^, Maciej Kopera^2^, Aleksandra Krasowska^2^, Aneta Michalska^2^, Hubert Suszek^4^, Andrzej Jakubczyk^2^, Marek Wypych^1^, Marcin Wojnar^2,5^, Artur Marchewka^1*^

^1^Laboratory of Brain Imaging, Nencki Institute of Experimental Biology of Polish Academy of Sciences, Warsaw, Poland

^2^Department of Psychiatry, Medical University of Warsaw, Warsaw, Poland

^3^Laboratory of Neurobiology of Vision, Nencki Institute of Experimental Biology of Polish Academy of Sciences, Warsaw, Poland

^4^Faculty of Psychology, University of Warsaw, Warsaw, Poland

^5^Department of Psychiatry, University of Michigan, Ann Arbor, MI, USA

*Corresponding authors:

Artur Marchewka, Ph.D, Associate Professor

Head of Laboratory of Brain Imaging (LOBI)

Nencki Institute of Experimental Biology

Polish Academy of Sciences

[a.marchewka@nencki.edu.pl](mailto:a.marchewka@nencki.edu.pl)

Jan Szczypiński MSc,

PhD Candidate at Laboratory of Brain Imaging (LOBI)

Nencki Institute of Experimental Biology

Polish Academy of Sciences

j.sczypinski@nencki.edu.pl

Full postal address:

Laboratory of Brain Imaging (LOBI), Nencki Institute of Experimental Biology, Polish Academy of Science, Pasteur 3, 02-093 Warsaw, Poland

| Supplementary Table S1. Physical properties of the stimuli in NCET. | | | | | |
| --- | --- | --- | --- | --- | --- |
| Photo filename | Width | Height | Luminance | Contrast | Entropy |
| b01.jpg | 1024 | 341 | 137.2616 | 40.0655 | 7.021 |
| b02.jpg | 1024 | 301 | 129.4396 | 53.8284 | 7.5269 |
| b03.jpg | 1024 | 300 | 132.5106 | 39.3517 | 7.0743 |
| b04.jpg | 1024 | 410 | 123.7202 | 63.8378 | 7.8084 |
| b07.jpg | 1024 | 361 | 138.388 | 51.7599 | 7.5521 |
| b09.jpg | 1024 | 345 | 112.9521 | 55.5572 | 7.5266 |
| b12.jpg | 1024 | 388 | 149.1558 | 57.4361 | 7.6417 |
| b13.jpg | 1024 | 342 | 182.8093 | 60.3856 | 7.4704 |
| b17.jpg | 1024 | 278 | 125.1086 | 51.7257 | 7.181 |
| b19.jpg | 1024 | 288 | 156.6417 | 58.4037 | 7.6471 |
| b22.jpg | 1024 | 349 | 171.2295 | 59.6545 | 7.5873 |
| b24.jpg | 1024 | 346 | 152.6566 | 58.5034 | 7.7524 |
| b30.jpg | 1024 | 375 | 166.1714 | 54.5141 | 7.5798 |
| b32.jpg | 1024 | 281 | 190.8793 | 50.8441 | 7.0947 |
| b36.jpg | 1024 | 363 | 141.366 | 58.4802 | 7.7147 |
| b37.jpg | 1024 | 278 | 161.9016 | 69.8258 | 7.8011 |
| b39.jpg | 1024 | 394 | 139.2658 | 51.4633 | 7.6178 |
| b44.jpg | 1024 | 356 | 148.8137 | 61.4221 | 7.7273 |
| g03.jpg | 1024 | 351 | 127.268 | 63.1027 | 7.7046 |
| g04.jpg | 1024 | 365 | 138.5679 | 59.5085 | 7.6024 |
| g06.jpg | 1024 | 317 | 134.5873 | 40.0327 | 6.9075 |
| g07.jpg | 1024 | 287 | 77.9375 | 70.8355 | 7.491 |
| g08.jpg | 1024 | 264 | 172.4925 | 57.1132 | 7.5739 |
| g10.jpg | 1024 | 341 | 151.7216 | 52.9018 | 7.4288 |
| g15.jpg | 1024 | 294 | 164.7007 | 63.5117 | 7.5928 |
| g16.jpg | 1024 | 384 | 166.7385 | 60.3598 | 7.629 |
| g21.jpg | 1024 | 335 | 152.2553 | 62.3117 | 7.7852 |
| g22.jpg | 1024 | 274 | 176.5164 | 51.2898 | 7.475 |
| g24.jpg | 1024 | 328 | 162.5011 | 80.172 | 7.6703 |
| g29.jpg | 1024 | 378 | 179.9103 | 68.6611 | 7.4404 |
| g35.jpg | 1024 | 397 | 139.6801 | 72.0297 | 7.8819 |
| g37.jpg | 1024 | 305 | 186.8343 | 53.8379 | 7.387 |
| g45.jpg | 1024 | 325 | 160.374 | 60.0898 | 7.7001 |
| g49.jpg | 1024 | 343 | 129.4185 | 52.9903 | 7.7063 |
| g50.jpg | 1024 | 332 | 152.2515 | 55.6325 | 7.5533 |
| g52.jpg | 1024 | 387 | 181.7438 | 49.652 | 7.3882 |

| Supplementary Table S2. Correlations between NCET, RMET and control measures. | | | | | | | |
| --- | --- | --- | --- | --- | --- | --- | --- |
|  | **Empathic Concern (IRI)** | **Personal Distress (IRI)** | **Percpective Taking (IRI)** | **PENN ER-40** | **TRS-S** | **Hinting Task Hints** | **Hinting Task Score** |
| **NCET** | -0.173 | -0.196 | 0.002 | 0.239 | -0.01 | 0.105 | -0.057 |
| **RMET** | 0.006 | 0.031 | -0.17 | **0.433*** | 0.043 | -0.046 | 0.009 |
| *NCET-Nencki Children Eyes Test; RMET - Reading the Mind in the Eyes Test; IRI - Interpersonal Reactivity Index; TRS-S Comprehension of Words Test Standard Version; * p < 0.05;* | | | | | | | |
|  |  |  |  |  |  |  |  |
